# Sleep tight! Adolescent sleep quality across three distinct sleep ecologies

**DOI:** 10.1093/emph/eoad040

**Published:** 2023-11-21

**Authors:** Andrea Silva-Caballero, Helen L Ball, Karen L Kramer, Gillian R Bentley

**Affiliations:** Institute of Anthropological Research, National Autonomous University of Mexico, Mexico City, 04510, Mexico; Department of Anthropology, Durham University, Durham, DH1 3LE, UK; Department of Anthropology, Durham University, Durham, DH1 3LE, UK; Department of Anthropology, Univesity of Utah, Salt Lake City, RM 4625, USA; Department of Anthropology, Durham University, Durham, DH1 3LE, UK

**Keywords:** adolescent sleep, sleep quality, sleep intensity hypothesis, sentinel hypothesis, sleep ecology, human evolution, human development

## Abstract

**Background and objectives:**

Good sleep quality, associated with few arousals, no daytime sleepiness and self-satisfaction with one’s sleep, is pivotal for adolescent growth, maturation, cognition and overall health. This article aims to identify what ecological factors impact adolescent sleep quality across three distinct sleep ecologies representing a gradient of dense urbanity to small, rural environments with scarce artificial lighting and no Internet.

**Methodology:**

We analyze variation of sleep efficiency, a quantitative measure of sleep quality—defined as the ratio of total time spent asleep to total time dedicated to sleep—in two agricultural indigenous populations and one post-industrial group in Mexico (Campeche = 44, Puebla = 51, Mexico City = 50, respectively). Data collection included actigraphy, sleep diaries, questionnaires, interviews and ethnographic observations. We fit linear models to examine sleep efficiency variation within and between groups.

**Results:**

We found that sleep efficiency varied significantly across sites, being highest in Mexico City (88%) and lowest in Campeche (75%). We found that variation in sleep efficiency was significantly associated with nightly exposure to light and social sleep practices.

**Conclusions and implications:**

Our findings point toward contextual cost-benefits of sleep disruption in adolescence. We highlight the need to prioritize research on adolescent sleep quality across distinct developmental ecologies and its impact on health to improve adolescent wellbeing through evidence-based health practices.

## INTRODUCTION

Building on the seminal studies of McKenna [1, 2] and Worthman [3, 4], who began studying sleep development using an anthropological, evolutionary theoretical framework, this article analyzes variation in sleep quality through sleep efficiency values (an objective dimension of sleep quality, defined as the ratio of total time spent asleep to total time dedicated to sleep) among adolescents in two agricultural indigenous and one post-industrial population in Mexico. These three contexts represent a gradient from a small, rural village having no Internet, or electronic device use, scarce artificial lighting and shared sleeping quarters, to a densely urban setting with outdoor and indoor artificial lighting, access to the Internet and screen-based devices. We explore which bio-socio-cultural environmental factors influence sleep quality, including how pubertal development, working, schooling, access to screen-based devices and electricity, daily exposure to natural light and social sleep behavior affect the sleep efficiency of adolescents. Critically, understanding sleep as an adaptive behavior and identifying which ecological factors promote or inhibit its quality are crucial for translating sleep research into a wider source of information that can be useful for producing evidence-based advice about optimal sleep for young people.

### Between deep and light sleep: revisiting the Sleep Intensity and the Sentinel hypotheses

Current inferences about the ecological history of human sleep have been drawn from comparisons with other mammals and among human societies with different means of subsistence (such as hunter–gatherers, agropastoralist groups and horticulturalists) [5–7]. After comparing the existing evidence for humans and other primates using phylogenetic analysis, Samson and Nunn (2015) identified four unusual traits that characterize human sleep on a gross level: (i) sleep is located in terrestrial and not arboreal environments, (ii) individuals engineer a ‘nest’ or sleeping platform for resting purposes, (iii) the human sleep duration is the shortest recorded among primates and (iv) human rapid eye movement (REM) sleep to non–REM (NREM) sleep ratio is the highest of all primates. Based on their observations, these authors proposed the Sleep Intensity Hypothesis, stating that early humans faced selective pressures that induced them to fulfill their sleep requirements in the shortest possible time [6]. A more efficient, shorter sleep would have benefited individuals by decreasing risks of predation and intergroup conflict in terrestrial environments, and increasing social interactions around sleeping times [7]. Furthermore, such changes in sleep would have also impacted human cognition, as extended active periods might have boosted the acquisition and transmission of new knowledge and skills, with deeper sleep also heightening their consolidation [6]. Nevertheless, more empirical research across human sleep ecologies is needed to prove or disprove this hypothesis.

Deep, intense sleep is characterized by high arousal thresholds [6, 8–10]. Importantly, these can be found in both, REM and NREM sleep, two states that alternate cyclically over an adult sleep episode. For example, during NREM sleep, encompassing stages N1, N2 and N3, arousal thresholds are lowest in N1-N2 and highest in N3 [6]. Meanwhile, during REM sleep, subclassified into tonic and phasic REM, arousal thresholds are lower in tonic sleep and higher in phasic sleep [9]. This means our sleep cycles also alternate between light and deep sleep [11, 12]. It has been proposed that such alternation serves a protective function since light sleep would enable the sleeping individual to waken quickly to screen the surrounding environment, detect potential danger and prepare for immediate flight or fight [13]. Therefore, the Sentinel Hypothesis, proposed by David Samson and colleagues, predicts that increased perceived risk of predation/insecurity leads to more frequent sleep arousals [14, 15]. This idea is grounded on sleep studies in numerous species noting that ecological factors, such as predation risk, foraging requirements and social life, shape patterns of sleep among mammals [16]. Yet, if the Sleep Intensity hypothesis is correct, early humans would have minimized nighttime risks via group size, chronotype variation, fire use and human engineering of sleep spaces, which, in turn, would have promoted deeper, more efficient sleep compared to other primates [6, 14, 17, 18].

However, relevant to understanding sleep’s role in human evolution and cognition, the Sleep Intensity and the Sentinel hypotheses have barely been tested cross-culturally in small-scale human populations [6, 14, 15]. Furthermore, most empirical sleep research in non-clinical contexts has been conducted solely in adult populations [19]. Hence, little is known about the role of the bio-socio-cultural ecology on sleep patterning, architecture and overall development, or its impact on sleep quantity, quality, memory consolidation, restorative functions and mental health cross-culturally [3]. Likewise, there is scarce systematic information on nonhuman primate sleep in the wild across ages [19]. Generating sleep data in ecologically relevant contexts will help understand the nuances of sleep and its related outcomes in human development, health and well-being.

### Sleep quality and sleep ecology: insights from anthropology

Sleep quality has been proposed as a practical measure to evaluate an individual’s restedness and an indicator of ‘optimal’ sleep function [20]. This measurement is central to testing the Sleep Intensity Hypothesis and the Sentinel Hypothesis. Good sleep quality is associated with slight sleep disturbances (expressed as short latency to sleep and sparse arousals), absence of daytime sleepiness, and self-satisfaction with the sleep experience [20, 21]. Notably, poor sleep quality during early adolescence plays an important role in the development of depression and anxiety disorders [22, 23]. Presumably, this is because sleep quality is linked to myelin regulation and is thus crucial to the development of the adolescent brain, which ends once the maturation of the dopaminergic reward system and the prefrontal cortex is completed [22, 23]. Moreover, NREM deep sleep has been linked to growth hormone (GH) secretion and the formation of immunological memory, with sleep fragmentation generally decreasing GH secretion and immune function [24, 25]. Therefore, sleep quality is crucial for adolescent growth, maturation and overall health.

Previous research examining sleep in contemporary adult hunter–gatherers, forest foragers, horticulturalists and rural communities with limited or no access to electricity, have reported sleep efficiency estimates between 66.4% and 88.7% [26–33], at times below the cut-off value of 85% for normal healthy sleep established in adult industrial populations [34] (86% for the case of adolescents [20]). Similarly, these non-clinical studies have reported wide variability in sleep durations, with averages ranging between 5.7 and 8.5 h/day [26–33, 35–39]. Additionally, it has been noted that insomnia complaints for initiating or maintaining sleep are rare in some of these groups, where local languages do not have a word for insomnia (e.g. the Tsimane and San people) [32, 38]. Existing evidence suggests that protected and comfortable environments shielded from sleep disruptors, along with co-sleeping habits, and group participation in economic activities, are factors that facilitate consolidated sleep bouts and homogeneous sleep activity among the social group [18, 33, 38].

The above findings question current ideal sleep parameters based on Western cultural assumptions and practices [40], as well as perceived wisdom asserting that compared to post-industrial societies, people in small-scale, rural or non-industrial contexts experience less fragmented (i.e. more efficient), longer bouts of sleep [26]. Nevertheless, empirical evidence of sleep quality in rural and non-industrial adolescent populations is limited given that research has focused chiefly on teen sleep duration and timing [18, 33, 41–46]. Comparative studies of sleep ecology are required to understand, not only the structural and cultural factors shaping adolescent sleep quality but also their potential interrelationship with functional and dysfunctional health outcomes [4, 47].

In examining variability of sleep quality of adolescents we predict that: (i) sleep efficiency values will be similar among the groups of youngsters in the three different ecological settings, (ii) nightly exposure to electric light (a well-documented sleep disruptor [48]) will decrease sleep efficiency, (iii) shorter adolescent sleep durations will be associated with higher sleep efficiency (i.e. fewer arousals), (iv) social sleep practices will increase sleep quality, (v) secluded sleep spaces shielded from environmental sleep disruptors will heighten sleep efficiency and (vi) more mature individuals will have higher sleep efficiency compared to early adolescents given that their sleep is characterized as having more frequent salient negative dreams and, consequently, higher arousability [49–51]. Testing these predictions across distinct human sleep ecologies also allows us to shed light on the Sleep Intensity Hypothesis (predictions i, ii and iii) and Sentinel Hypothesis (predictions iv, v and vi), therefore contributing to current understanding about the natural history of human sleep.

## METHODOLOGY

### Study locations and participants

We worked with 163 participants (females = 48%) between 11 and 16 years old (mean age 13.7, SD ± 1.21) recruited from local schools in three sites in Mexico in 2019: (i) a densely populated, post-industrial urban center (Mexico City, *n* = 67, February 1st to April 8th), (ii) an indigenous Totonac agricultural town (San Juan Ozelonacaxtla, Puebla, *n* = 51, 6th to November 11th), and (iii) a small-scale Maya agricultural village (Xculoc, Campeche, *n* = 45, May 31st to July 5th). Adolescents participated in the study over 10 continuous days, comprising six school days and four free (non-school) days.

The three distinct study locations (henceforth called Mexico City, Puebla and Campeche, respectively) were chosen following four main criteria to allow ecological comparisons of adolescent sleep (see Table 1 for sites’ characteristics): (i) Rural populations should belong to small-scale societies, relatively isolated geographically, socially and/or culturally, without ready access to electricity or electronic devices. (ii) The urban population should belong to affluent, educated households, depending on industrial/post-industrial economies, living in densely populated areas with ready access to electricity or electronic devices. (iii) Each site had to have an established gatekeeper (i.e. fellow researchers) who was contacted before the start of the study to ease communication with local populations. (iv) All three sites had to be located outside high-risk areas of Mexican territory according to the international advisory travel guidelines (https://travel.state.gov/content/travel/en/traveladvisories/traveladvisories/mexico-travel-advisory.html, https://www.gov.uk/foreign-travel-advice/mexico).

**Table 1.** Characteristics of the study locations

Site characteristics		
**Mexico City**	**Puebla**	**Campeche**
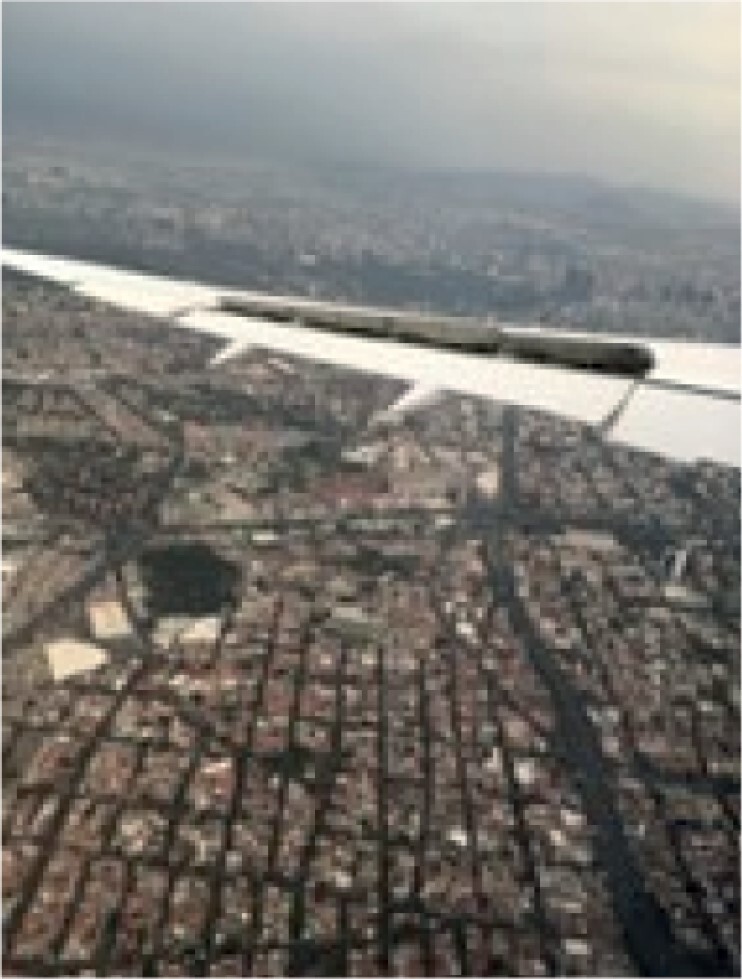	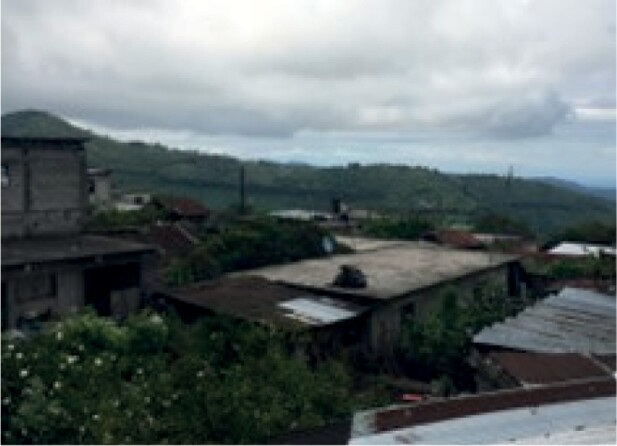	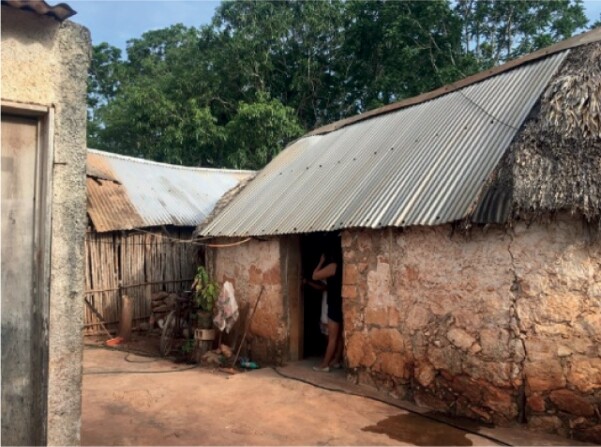
*Geographic location*		
19.42847, -99.12766 The city is located in a highlands plateau, 2,303 m above sea-level.	20.183333, -89.833333 The town lies 834 m above sea-level at the intersection of two mountain systems.	20.046944, -97.613611 The village is located in the Yucatan Peninsula plain, 69 m above sea level.
*Economy*		
Post-industrial dense urban center with a largely service-based economy. Participants inhabit the South and Northeast urban areas of Mexico City. They are from reasonably well-off, educated households whose members work in a variety of professions and trades, including accountants, engineers, teachers, doctors and dentists, among others.	Totonac farming town experiencing radical infrastructural changes since the end of the 1980s. Firstly with the introduction of electricity and running water and then with the construction of schools, better roads and a neighborhood health center. Traditional farming techniques are still used due to regional orographic characteristics preventing the adoption of mechanized agriculture. The town’s household economies rely on agriculture, animal breeding, trades, and remittances from migrant family members. Children and adolescents with migrant parents typically stay in the community under the care of close relatives.	Small agricultural Maya village undergoing demographic and economic transitions due to the recent introduction of running water, electricity, new farming techniques promoting intensive agriculture, schools and roads. Despite these developments, the village remains isolated from public transport. Household economies mainly rely on subsistence agriculture and crop sales, including peanuts, corn, and squash, animal breeding consisting of ducks, chicken, and sheep, apiculture, construction labor or work in neighboring village restaurants.
*Inhabitants and ethnic composition*		
~8.9 million inhabitants Admix ethnic background encompassing ‘mestizo’ (89%), indigenous (9%) and Afro-descendant (2%) populations.	~1,368 inhabitants Population in San Juan Ozelonacaxtla identify as Totonac, Mexico’s tenth largest ethnic group.	~835 inhabitants Population in Xculoc identify as ‘Mayeros’ or Maya, Mexico’s second largest ethnic group.
*Main language*		
Spanish	Highland Totonaku	Peninsular Maya

Following a verbal explanation of the study’s purpose and having read the study consent form, participants and their parents gave written, signed agreement to participate. With the assistance of local helpers fluent in Highland Totonaku and Spanish or Peninsular Maya and Spanish, valid consent was secured. The Ethics Committee of Durham University’s Department of Anthropology approved the study protocol. All procedures followed the Declaration of Helsinki and the standards established by the Mexican General Health Regulation on Health Research Matters and the Mexican Official Standard NOM-012-SSA3-2012 for implementing health research projects in Mexico.

### Data collection

We used wrist actigraphy, sleep diaries, semi-structured interviews, ethnographic observations and a standardized questionnaire to gather data on participants’ sleep characteristics (including sleep timing, duration and efficiency), environmental factors (such as exposure to seasonal variations in temperature and light, access to electricity, use of electronic devices, bedding characteristics and social sleeping practices), pubertal maturation and daily activities. To ensure data quality, all data collection instruments were verbally explained to participants and any questions answered.

#### Actigraphy.

Data on sleep behavior, daily physical activity and ambient light were collected via Motionlogger Micro Watch units (Ambulatory Monitoring Inc., USA) using 1-min epochs set in the Zero Crossing Mode. A total of 147 participants wore the actigraphy devices over 10 days (six workdays and four free days) for 24 h. They were instructed to use the event logger button prior to any sleep event throughout the study.

Due to watch malfunctions, the final sample was reduced to 145 adolescents (72 females) with valid actigraphy data (Mexico City = 50, Puebla = 51, Campeche = 44). We compiled 1405 sleep observations where 618 corresponded to non-school nights and 787 to school nights. Wrist actigraphy measurements were further validated against sleep diaries and data from semi-structured interviews to detect ‘false’ wake-like or sleep-like entries [33, 52]. The actigraphy sleep data were analyzed using the Sadeh algorithm with ActionW 2.7 software.

#### Sleep diary and questionnaire.

 Data on sleep behavior and daytime drowsiness were attained using a customized 10-day sleep diary based on the Consensus Sleep Diary and the Paediatric Daytime Sleepiness Scale [53, 54]. A straightforward design with scales, graphics, and colors allowed completion by participants who had difficulty reading or writing. Adolescents were asked to fill in their diary before and after their nightly sleep. To explore whether their sleep patterns were associated with pubertal development or stemmed from an interaction effect, every participant responded to the Pubertal Development Scale (PDS), a standardized instrument that provides non-invasive assessment of pubertal status [55].

#### Interviews and observation.

 Information about the sleep environment of each adolescent was obtained through semi-structured interviews of 45 min on average. Interviews were aimed at addressing: (i) current sleep–wake patterns and sleep quality, (ii) access to electricity and/or electronic devices, (iii) characteristics and cultural settings in which participant slept and (iv) cultural ideas and practices regarding sleep [56]. Ethnographic observations within and outside the schools further explored adolescents’ peer relationships, social roles, daily lifestyles and attitudes toward sleep. A field diary was kept documenting daily observations, activities, informal conversations, remarks on interviews and annotations about research development.

#### Calculation of solar and meteorological variables.

 Data on sunrise and sunset times were obtained through a solar geometry study based on Iqbal (1983) [57]. Additionally, each site’s geographical coordinates were mapped with the NASA Langley Research Center (LaRc) POWER Project (https://power.larc.nasa.gov/) to obtain daily data on temperature, sky insolation incident, the Insolation Clearness Index, precipitation, humidity and surface pressure.

### Data analysis

Statistical analyses were performed in R version 3.6.3; statistical inferences were ascertained from *P*-values (significance levels set at 0.05) and confidence intervals. Descriptive statistics characterized sleep measurements in our sample population (Table 2). To test our predictions, we primarily focused our analyses on the variation of participants’ sleep efficiency values. Further data on adolescent sleep duration are presented elsewhere [46]. We employed a two-tailed Wilcoxon Rank Sum Test to gauge if differences in participants’ sleep efficiency between school and non-school nights were significant. We ran a two-tailed Kruskal–Wallis test to evaluate specific differences between sites.

**Table 2.** (a) Sample composition at each site. (b) Micro- and Macro-ecological characteristics of participants’ sleeping sites.

(a) Sample characteristics		Mexico City	Puebla	Campeche
Sex (n)	Girls	24	27	21
	Boys	26	24	23
Age	Mean (SD)	13.8 (1.3)	13.5 (1.1)	14 (1.3)
	Min-Max	11.2–16.5	11.8–15.8	11.6–16.7
Weight status %	Underweight	0	6	2
	Normal weight	80	76	80
	Overweight	16	16	18
	Obese	4	2	0
Pubertal Development Stage %	Pre/Early puberty	20	14	23
	Mid puberty	22	31	29
	Advanced puberty	58	55	48
**(b) Sleep ecology**		**Mexico City**	**Puebla**	**Campeche**
Access to screen-based devices %^a^	Mobile phones	94	67	57
	Tablets	54	35	9
	Computers	94	18	9
	TV	100	96	98
Darkness/ Light %	Dark	76	39	68
	External illumination ^b^	18	24	16
	Indoors light	6	37	16
Sleeping arrangements %	Solitary sleep	72	33	11
	Social sleep^c^	28	67	89
Co-sleepers’ generation %^d^	Children/ adolescents	88	53	47
	Adults	12	21	8
	Both	0	26	45
Materiality of sleep	Sleeping surface	Bed	Bed or traditional wooden surface	Hammock
	Housing materials	Concrete, steel, and bricks	Concrete, steel, bricks, rocks, wood and cardboard	Concrete, steel, bricks, raw earth, palm, wood and cardboard
School demands	School day start	7:30 h	8:00 h	8:00 h
	Commuting times	5–50 min^e^	5–10 min^f^	5–10 min^f^

Data are represented as percentages relative to each sample group.

^a^Adolescents in Puebla and Campeche have limited access to screen-based devices compared to those from Mexico City. Most borrow these devices from family members instead of owning them like urban participants.

^b^Streetlights or moonlight are considered as external sources of night lighting.

^c^Social sleep includes room- and bed-sharing practices.

^d^Percentages are relative to the total amount of participants practicing social sleep in each site.

^e^By walk, car, public transport or school bus.

^f^By walk.

To investigate the effects of sexual maturation, temperature, artificial and natural light and co-sleeping practices on participants’ sleep, we used the *lme4* R-package to fit a generalized multilevel model with ‘subject’ and ‘site’ as random effects [58]. To further analyze the specific impacts of our predictor variables on participants’ sleep at each location, we ran three two-level site-specific models. These models incorporated ‘subject’ as a random effect to control for repeated measures nested within individuals. Since the sleep efficiency values had a non-normal, left skewed distribution, which hindered statistical analysis, the multilevel analyses incorporated sleep inefficiency (an opposite value to sleep efficiency with a skewed-to-the-right distribution) as the outcome variable employing a Poisson distribution.

Each model tested pubertal maturation (characterized as pre/early, mid and advanced puberty), gender (F/M), daytime napping (Y/N), sleep midpoint (i.e. the halfway point between sleep start and sleep end), nightly light exposure (the percentage lux count epochs relative to an individual’s sleep duration), sunrise times, temperature, co-sleeping practices (room-sharing, bed-sharing or none), co-sleepers’ generation (the co-sleeper’s status as a child or an adult individual), assisted awakening (the use of an external agent, alarm or person, to wake up) and day of the week (school day/free day) as predictor variables (full models are in Supplementary Tables 1 and 2). To fit and select our final models, we employed the AIC criteria with the *MuMin* package [59]. All the residuals of the best-fitting models had normal distributions.

## RESULTS

### Sleep ecology

Table 2 characterizes participants’ traits at each site, as well as the ecological settings where they slept. The three study locations reflect a gradient between densely urban and more rural, subsistence-based lifestyles in terms of access to technology and sleeping conditions. We observed marked differences in adolescents’ exposure to media and the Internet, their use of indoor and outdoor artificial lighting, whether they slept alone or with other family members, characteristics of their sleeping quarters and their school and chore routines.

While most urban adolescents reported sleeping in dark environments (88%), around 47% of rural participants reported sleeping with the lights on or being illuminated by streetlights or moonlight. A high proportion (37%) of Totonac respondents in Puebla reported keeping a light on while sleeping, and 24% had an external light source. Participants explained that they utilized an illumination source while sleeping because the dark scared either them or a family member or that they kept their cell phone/tablet/television on because watching images helped them to fall asleep.

The architectural characteristics of sleeping quarters alongside school demands also varied along the gradient of highly urban to more rural, subsistence-based environments. Urban adolescents mostly practiced solitary sleep within spaces devised to buffer environmental sound, light, temperature and wind oscillations, while rural adolescents traditionally slept in shared quarters (with children, adults or both) that provided less effective buffering from environmental cues (see Table 2b). Exposure to external cues during sleeping was most evident in Campeche.

Regarding school demands, there were steep differences between urban and rural schooling systems given that Mexican rural education varies according to local State/Municipal assets and the restraints and expectations posed by the respective communities. As a result, the educational system is loosely regulated in Campeche, more structured in Puebla, and fairly rigid in Mexico City, where students must adhere to stringent schedules and high workloads.

### Sleep efficiency and associated bio-socio-cultural factors

Table 3 summarizes sleep characteristics for each study site. On school nights, adolescents in Mexico City expressed the shortest sleep duration (471 min, SD = 58 min) and the highest sleep efficiency (88%, IQR = 7). In contrast, Maya adolescents in Campeche expressed the shortest sleep duration during free nights (535 min, SD = 89 min), and had the lowest sleep efficiency (75%, IQR = 13). Although sleep times and duration varied significantly between school nights and free nights, statistical difference was not found for sleep efficiency values (W = 183,059, *P* = 0.35, *r* = −0.027) even though there were significant differences across the three study sites for sleep efficiency (H(2) = 295.31, *P* < 0.001). Specifically, urban participants had the highest scores and rural participants in Campeche the lowest. Differences between weekdays and weekends across the three sites suggest that each adolescent population responded differently to their own time constraints.

**Table 3.** Values for sleep times and duration by sites

Weeknight	Site	Start time	End Time	Sleep duration	Sleep efficiency	Nap ratio
School days	Mexico City	22:23 (54.58)	6:12 (27.12)	471.01 (57.94)	88 [7]	17.23 (22.04)
	Puebla	22:31 (65.94)	6:56 (28.45)	508.41 (65.36)	84 [9]	13.07 (18.05)
	Campeche	22:48 (47.33)	6:56 (29.56)	488.66 (50.18)	78 [14]	20.12 (27.43)
Free nights	Mexico City	23:30 (82.11)	8:45 (93.61)	556.47 (91.39)	87 [8]	14.78 (19.78)
	Puebla	22:33 (73.3)	8:01 (74.99)	571.06 (88.16)	85 [7]	13.56 (19.79)
	Campeche	23:05 (80.01)	7:59 (65.58)	535.39 (89.16)	75 [13]	18.75 (26.85)

Data are represented as mean (standard deviation) and median [interquartile range]. Standard deviation is expressed in minutes for sleep times and sleep duration values. Sleep duration is defined as the interval between when the participant goes to bed/wooden surface/hammock to sleep (sleep start), and the time she/he gets out of the sleeping surface (sleep end). Nap ratio is defined as the proportion of actigraphy-assessed nap days, calculated as the total number of days with at least one observed nap divided by the number of days with actigraphy data. We scored a period of inactivity in our actigraphy data as time in bed/wooden surface/hammock if it exceeded 210 min or as a nap episode if it fell within the threshold of 15–210 min and separate by at least an hour from a main big sleep [27].

Our final general model examining biological and ecological influences on sleep incorporated gender, sleep midpoint, dim ambient light at night (<20 Lux), sunrise times and the interaction of co-sleeping practices and co-sleepers’ generation (CP:CG) as predictor variables for the sleep inefficiency variance (*R*^2^_Marginal/ Conditional_ = 0.086/0.705; ICC_Adjusted/ Conditional_ = 0.677/0.619) (Table 4). The incidence rate of arousal per sleep bout was negatively associated with girls, sleep-midpoint (measurement encapsulating sleep start and end times), and the practice of room/sharing with adults. In contrast, arousal incidence rate was positively associated with nightly exposure to dim light and sunrise times. It was therefore more likely to observe greater sleep efficiency in girls, adolescents with later sleep times and individuals sharing rooms with adults, and lower efficiency in participants exposed to artificial or natural light while sleeping.

**Table 4.** Bio-socio-cultural predictors of sleep inefficiency variation (i.e. the ratio of total time spent awake to total time dedicated to sleep) across locations

Predictors	Incidence rate ratios	CI	*P*	df
Gender ^a^	0.84	0.76–0.92	**<0.001**	1179
Sleep midpoint	1.00	1.00–1.00	**0.004**	1179
Nightly exposure to light (20lux)	1.01	1.00–1.01	**0.001**	1179
Sunrise times	1.00	1.00–1.00	**<0.001**	1179
Solitary sleep	0.91	0.74–1.11	0.343	1179
Room sharing w/children	0.94	0.77–1.14	0.517	1179
Bed sharing w/children	1.07	0.83–1.38	0.595	1179
Room sharing w/adults	0.72	0.55–0.93	**0.012**	1179
Bed sharing w/adults	1.12	0.80–1.57	0.522	1179
Room sharing w/both	1.01	0.81–1.27	0.915	1179
*Sites (Intercept)*				
Mexico City	−0.24			
Puebla	−0.03			
Campeche	0.29			

Coefficients below 1 indicate a higher incidence rate of continuous sleep per sleep bout, while coefficients over 1 a higher incidence rate of arousal.

^a^Boys are the reference category for gender; the estimates are for girls.

Regarding the site-specific models (Table 5), the final model for Mexico City comprised gender, sleep-midpoint, dim ambient light at night, assisted awakening and the CP:CG interaction as predictor variables for sleep inefficiency (*R*^2^_Marginal/Conditional_ = 0.152/0.569; ICC_Adjusted/Conditional_ = 0.492/0.417). The arousal rate per sleep bout was negatively associated with girls, sleep-midpoint, assisted awakening and solitary sleep, and positively associated with nightly light exposure. The model for Puebla included weeknight, gender, sleep-midpoint, naps before the main sleep, nightly light exposure, sunrise timing, temperature and the CP:CG interaction as predictor variables (*R*^2^_Marginal/Conditional_ = 0.225/0.567; ICC_Adjusted/Conditional_ = 0.441/0.342). Here, the arousal incidence rate was negatively associated with girls, sleep-midpoint, and room-sharing with adults, and positively associated with school nights, napping behavior, nightly light exposure and sunrise timing. Finally, the model for Campeche incorporated pubertal maturation, gender, sunrise timing and temperature as predictor variables (R^2^_Marginal/ Conditional_ = 0.180/0.666; ICC_Adjusted/Conditional_ = 0.593/0.486). Arousal incidence rate in Campeche was negatively associated with mid-puberty and girls, while positively associated with sunrise times.

**Table 5.** Bio-socio-cultural predictors of sleep inefficiency variation for each study site

Site-specific final models	Mexico				Puebla				Campeche			
Predictors	Incidence Rate Ratios	CI	*P*	df	Incidence Rate Ratios	CI	*P*	df	Incidence Rate Ratios	CI	*P*	df
Sleep midpoint	1	1.00–1.00	**<0.001**	408	1	1.00–1.00	**0.002**	403				
Gender ^a^	0.85	0.72–1.01	0.058	408	0.89	0.77–1.03	0.123	403	0.68	0.52–0.89	**0.005**	351
Nightly exposure to light (<20lux)	1.01	1.01–1.02	**<0.001**	408	1.11	1.05–1.17	**<0.001**	403				
Assisted awakening	0.84	0.77–0.91	**<0.001**	408								
Solitary sleep	0.64	0.42–0.96	**0.031**	408	1.06	0.83–1.35	0.635	403				
Room sharing w/children	0.77	0.50–1.19	0.238	408	1.04	0.81–1.35	0.749	403				
School night					1.06	1.00–1.13	**0.041**	403				
Nap before main sleep					1.11	1.03–1.19	**0.008**	403				
Sunrise timing					1.002	1.00–1.00	**<0.001**	403	1.001	1.00–1.00	**0.005**	351
Minimum temperature (°C)					0.99	0.97–1.00	0.1	403	0.98	0.95–1.01	0.138	351
Bed sharing w/children					1.17	0.88–1.55	0.276	403				
Room sharing w/adults					0.68	0.51–0.92	**0.011**	403				
Bed sharing w/adults					0.92	0.61–1.37	0.674	403				
Room sharing w/both					0.72	0.51–1.03	0.069	403				
Mid puberty									0.76	0.61–0.95	**0.016**	351
Advanced puberty									1.02	0.78–1.34	0.885	351

Coefficients below 1 indicate a higher incidence rate of continuous sleep per sleep bout, while coefficients over 1 a higher incidence rate of arousal.

^a^Boys are the reference category for gender; the estimates are for girls.

## DISCUSSION

We found support for three of our six predictions, since sleep efficiency was significantly different across groups and was modulated by nightly light exposure, the degree of environmental isolation of sleeping spaces, social sleep arrangements and advanced pubertal development, but not by sleep duration. The lack of straightforward relationships between low sleep efficiency, access to personal electronics and changes in sleep duration suggests that the impact of ecological factors on sleep measurements is more complex than commonly appreciated. As discussed further in this section, our results point toward culture having a significant role in sleep variation beyond a community’s economic and developmental status.

### Sleep quality and sleep duration

Although we expected to find similar sleep efficiency values among the groups of teenagers in the three distinct ecological settings, we identified significant differences across sites. Notably, compared to sleep efficiency in Mexico City (88%) and Puebla (85%), teenagers in Campeche (75%) presented clear sleep efficiency values below the cut-off value of 86% for normal healthy sleep established in adolescent industrial populations [20] despite having limited access to personal electronics at night. After controlling for pubertal development and gender, differences in sleep duration were not statistically associated with changes in sleep efficiency. Even though sleep durations were strikingly similar in Mexico City and Campeche [46], sleep efficiency at these two sites differed substantially, with rural indigenous adolescents in Campeche spending 10–12% more time awake during their nightly sleep compared to urban teenagers in Mexico City.

Prior studies examining ecological constraints of sleep architecture have described that sleep duration increases when fragmented [19, 60]. Therefore, we had hypothesized that shorter sleep durations would be associated with higher adolescent sleep efficiency. However, our results did not support this idea. Furthermore, the finding that sleep efficiency varied, not only between rural and urban contexts, but also among both rural groups, suggests that sleep efficiency is a plastic trait, highly responsive to socio-cultural ecological influences.

### Consequences of nighttime lights, housing and sociability on sleep

We confirmed nightly exposure to light as a degrader of adolescent sleep. Except for the Campeche model, all best-fitting models indicated that teenagers exposed to artificial/natural light while sleeping were more likely to exhibit lower sleep efficiency values. The apparent uniqueness of Campeche, the most ‘traditional’ location in our study, might arise from local sleeping practices, as teenagers generally slept in dark conditions and had restricted access to screen-based gadgets, cell phones and the Internet.

While nightly exposure to light partially explained differences in adolescent sleep efficiency, substantive differences across sites likely stemmed from variations in the environmental hazards perceived by the sleepers, as well as their social sleep arrangements. In this sense, results suggested that human-engineered spaces meant to shield sleep from environmental changes heightened sleep efficiency [61]. The notion that such sleeping conditions decreased the perceived risk of predation/insecurity, thereby reducing sleep arousals [15], accords with the observed variation of adolescent sleep efficiency across sites, since sleep in Campeche was less buffered from biotic/abiotic environmental cues, more protected in Puebla and effectively shielded in Mexico City.

Additionally, results partially support the idea that social sleep practices increase sleep quality, highlighting the role of the co-sleepers’ age group and cultural norms on adolescent sleep quality. For instance, while participants sleeping with adults in Puebla were more likely to have a higher sleep efficiency (probably because adolescents feel safer when close to adults at night, as suggested by interviews), adolescents practicing solitary sleep in Mexico City had higher efficiency values. Meanwhile, no such effect was found in Campeche. These contrasting findings likely result from distinct sleep cultures across sites, as solitary sleep was predominant in Mexico City (72%), whereas sharing the sleep quarters with all the household members was a dominant custom in Campeche (89%). In comparison, high variability regarding social sleep practices was observed in Puebla, with some practicing solitary sleep (33%) and others sharing their sleeping spaces with adults, children or both (67%). Therefore, it is reasonable to state that Puebla represented an ideal scenario to explore how distinct social sleep practices influence adolescent sleep quality.

### Developmental stage and sleep outcomes

We found no support for the idea that immature adolescents have lower sleep efficiency due to frequent salient negative dreams and, consequently, increased arousability [49–51]. Instead, results from our full general and site-specific models pointed toward the opposite trend, suggesting decreased sleep efficiency from pre/early to advanced puberty. Furthermore, mid-pubertal teenagers in Campeche were statistically more likely to have higher sleep efficiency values than early and mature adolescents.

Developmentally related differences in sleep efficiency might reflect bio-psycho-social changes associated with pubertal stages. In this sense, it is possible that, compared to pre/early pubertal participants, more mature individuals experienced more sleep arousal episodes due to changes in homeostatic mechanisms regulating sleep pressure (expressed as a less robust build-up and decay of sleep pressure), the quality and quantity of social bonds out of the household, social responsibilities (such as farming or house work), freedom to set one’s own bedtimes and shifts in cognitive-emotional states due to ongoing development and maturation of the dopaminergic reward system and prefrontal cortex [8, 18, 22, 62, 63]. A decreased sleep efficiency might serve a protective function for the social group, enabling more mature individuals, better prepared than younger individuals to physically respond to nocturnal environmental threats, to monitor the surroundings at night.

Remarkably, differences in sleep efficiency across and within sleep ecologies might also reflect differences in sleep architecture relative to the REM to NREM sleep ratio. Since sleep architecture correlates with cognitive development, memory consolidation, restorative functions, energy conservation and affective processing [4, 17, 64, 65], we underscore here the pressing need to conduct further comparative studies of sleep ecology to understand not only the structural and cultural factors shaping adolescent sleep quality but also their potential interrelationship with different NREM and REM duration trajectories across human development.

### Implications for the Sleep Intensity and Sentinel Hypotheses

By providing evidence in favor of adolescent sleep efficiency being an adaptive phenotypic trait, results align with the premises of the Sentinel Hypothesis while challenging the notion that human sleep is characterized by short, deep sleep, as purported by the Sleep Intensity Hypothesis. Nevertheless, it should be noted that while examining sleep in small-scale human populations across ages, climates, and latitudes is critical to test the Sleep Intensity Hypothesis, more information on sleep efficiency variation at the interspecies level is required to support or refute this hypothesis [6]. If there were quality data showing that, compared to other apes, human sleep is less efficient, then the Sleep Intensity Hypothesis would be unquestionably disproved. Unfortunately, due to the intrinsic difficulties of studying non-human sleep in evolutionary-relevant environments (i.e. non-captive conditions) [16], current available comparative data on the sleep efficiency of our closest phylogenetic relatives are scarce. To our knowledge, there are only three papers to date that have used actigraphy or video recordings to examine non-human primate sleep efficiency in outdoor captivity or non-captive conditions [17, 66, 67]. These reported mean sleep efficiency values of 59% and 85% for baboons (*Papio papio* and *Papio anubis*, respectively), 73% for Orangutans (*Pongo spp*.) and 86% for Chimpanzees (*Pan troglodytes*).

Our findings here add to the currently limited empirical evidence of sleep quality in rural and non-industrial adolescent populations given that research so far has focused chiefly on teen sleep duration and timing [18, 33, 41–46]. Consistent with previous research examining adult sleep in communities with limited or no access to electricity [26–33], this study results call into question conventional wisdom and current ideal sleep parameters based on Western cultural assumptions and practices [40], stating that people living in small-scale, non-industrial or rural contexts sleep for longer and experience less fragmented sleep than those in post-industrial societies [26].

### Strengths and limitations

The diverse ethnic and socioeconomic backgrounds of the participants are the main strength of this study since the results of sleep studies in naturalistic settings are difficult to compare with those of urban or lab environments due to different protocols and methodologies. However, our research has some limitations: (i) although we sampled 87% of the available people who fit the sample criteria in both indigenous, rural communities, convenience sampling resulted in a small sample size; (ii) we lacked longitudinal data for comparing sleep across age groups and determining sleep developmental trajectories; (iii) likewise, we could not include information on seasonal differences in nutrition, energy use, and allocation, which may affect sleep characteristics; (iv) compared to Tanner pubertal staging, the PDS, a self-reported tool for assessing pubertal state, may not accurately reflect the specific developmental stage of teenagers.

## CONCLUSION

This work highlights sleep efficiency as phenotypically plastic and adaptive. Our results indicate that consolidated, short, deep sleep is pervasive in modern, industrial/post-industrial sleep environments. Furthermore, this study provides evidence that safe sleep shelters and social sleep practices facilitate higher sleep quality, where more mature adolescents (more physically able to respond to nightly environmental threats than younger individuals) present higher sleep arousability. These findings question the notion that short, deep sleep is a hallmark of human sleep, challenging the Sleep Intensity Hypothesis, and providing evidence in favor of the Sentinel Hypothesis. Overall, our findings point toward contextual cost-benefits of sleep disruption in adolescence and contribute to the field of comparative developmental ecology and to unraveling the natural history of sleep.

## Supplementary Material

eoad040_suppl_Supplementary_Material

## Data Availability

The datasets created and used in the current study are publicly accessible under the title Daily adolescent sleep, Mexico 2019_Dataset (doi:10.15128/r16682x402c) in the Durham Research Online DATAsets Archive (DRO-DATA).
