# Supplementary material for: Sleep tight! Adolescent sleep quality across three distinct sleep ecologies
Source: Evol Med Public Health. 2023 Nov 21;11(1):448–60. doi: 10.1093/emph/eoad040 (PMC10693291; doi:10.1093/emph/eoad040)
Supplement: eoad040_suppl_Supplementary_Material [file eoad040_suppl_supplementary_material.pdf]

Supplementary Table 1. Resulting all-sites full model

**All-sites full model**				
Predictor	*Incidence Rate Ratios*	*CI*	*p*	*df*
School night	0.99	(0.96, 1.03)	0.585	1176
Mid puberty	0.9	(0.78, 1.05)	0.177	1176
Advanced puberty	1.03	(0.90, 1.18)	0.638	1176
Gender [a]	0.78	(0.68, 0.88)	**<0.001**	1176
Sleep midpoint	1	(1.00, 1.00)	**0.004**	1176
Nightly exposure to light (20lux)	1.01	(1.00, 1.01)	**0.001**	1176
Sunrise times	1	(1.00, 1.00)	**<0.001**	1176
Solitary sleep	0.94	(0.76, 1.15)	0.549	1176
Room sharing w/children	0.96	(0.79, 1.18)	0.72	1176
Bed sharing w/children	1.12	(0.87, 1.44)	0.386	1176
Room sharing w/adults	0.74	(0.57, 0.95)	**0.02**	1176
Bed sharing w/adults	1.16	(0.83, 1.62)	0.39	1176
Room sharing w/both	1.03	(0.82, 1.28)	0.822	1176
Sites (Intercept)				
Mexico City	0.77			
Puebla	0.97			
Campeche	1.3			

[a] Boys are the reference category for gender; the estimates are for girls

Supplementary Table 2. Resulting full models for Mexico City, Puebla and Campeche

**Site-specific full models**	**Mexico City**				**Puebla-Totonac**				**Campeche-Maya**			
Predictors	*Incidence Rate Ratios*	*CI*	*p*	*df*	*Incidence Rate Ratios*	*CI*	*p*	*df*	*Incidence Rate Ratios*	*CI*	*p*	*df*
School night	1	(0.88, 1.13)	0.949	402	1.09	(1.01, 1.17)	0.036	400	0.96	(0.91, 1.02)	0.161	341
Mid puberty	1.04	(0.81, 1.34)	0.764	402	1.02	(0.78, 1.35)	0.867	400	0.77	(0.63, 0.94)	**0.012**	341
Advanced puberty	1.19	(0.96, 1.47)	0.117	402	1.15	(0.90, 1.46)	0.27	400	1.08	(0.82, 1.43)	0.57	341
Gender [a]	0.79	(0.65, 0.96)	**0.019**	402	0.84	(0.71, 1.00)	**0.056**	400	0.62	(0.46, 0.82)	**0.001**	341
Sleep midpoint	1	(1.00, 1.00)	**0.023**	402	1	(1.00, 1.00)	**0.001**	400	1	(1.00, 1.00)	0.929	341
Nap before big sleep	1	(0.92, 1.09)	0.99	402	1.11	(1.03, 1.19)	**0.008**	400	0.97	(0.91, 1.04)	0.449	341
Nightly exposure to light (<20lux)	1.01	(1.01, 1.02)	**<0.001**	402	1.11	(1.05, 1.17)	**<0.001**	400	1	(0.99, 1.00)	0.388	341
Sunrise timing	1	(1.00, 1.00)	0.976	402	1	(1.00, 1.00)	**<0.001**	400	1	(1.00, 1.00)	0.31	341
Minimum temperature (°C)	1.01	(0.99, 1.04)	0.289	402	0.99	(0.97, 1.00)	0.116	400	0.98	(0.95, 1.01)	0.11	341
Assisted awakening	0.84	(0.73, 0.95)	**0.007**	402	0.96	(0.88, 1.05)	0.383	400	1.02	(0.96, 1.08)	0.475	341
Solitary sleep	0.6	(0.40, 0.90)	**0.014**	402	1.04	(0.81, 1.33)	0.772	400	1.08	(0.79, 1.48)	0.611	341
Room sharing w/children	0.72	(0.47, 1.11)	0.135	402	1.03	(0.80, 1.34)	0.795	400	0.9	(0.70, 1.17)	0.433	341
Bed sharing w/children	N/A	N/A	N/A	N/A	1.13	(0.84, 1.51)	0.418	400	1.2	(0.84, 1.70)	0.315	341
Room sharing w/adults	N/A	N/A	N/A	N/A	0.66	(0.49, 0.89)	**0.007**	400	0.87	(0.62, 1.22)	0.423	341
Bed sharing w/adults	N/A	N/A	N/A	N/A	0.89	(0.59, 1.34)	0.581	400	N/A	N/A	N/A	N/A
Room sharing w/both	N/A	N/A	N/A	N/A	0.67	(0.47, 0.96)	**0.03**	400	1.18	(0.89, 1.57)	0.237	341

[a] Boys are the reference category for gender; the estimates are for girls
